# Diet Supplementation with Polyphenol-Rich *Salicornia ramosissima* Extracts Protects against Tissue Damage in Experimental Models of Cerebral Ischemia

**DOI:** 10.3390/nu14235077

**Published:** 2022-11-29

**Authors:** Paula García-Rodríguez, Feifei Ma, Carmen del Río, Marina Romero-Bernal, Ana M. Najar, María de la Luz Cádiz-Gurrea, Francisco Javier Leyva-Jimenez, Laura Ramiro, Paloma Menéndez-Valladares, Soledad Pérez-Sánchez, Antonio Segura-Carretero, Joan Montaner

**Affiliations:** 1 Neurovascular Research Laboratory, Vall d’Hebron Institute of Research (VHIR), Universitat Autònoma de Barcelona, Hospital Universitari Vall d’Hebron, Pg. Vall d’Hebron 119-129, 08035 Barcelona, Spain; 2 Institute of Biomedicine of Seville (IBiS), Hospital Universitario Virgen del Rocío, CSIC, Universidad de Sevilla, 41013 Seville, Spain; 3 Department of Neurology, Hospital Universitario Virgen Macarena, 41009 Seville, Spain; 4 Department of Analytical Chemistry, University of Granada; Av. Fuentenueva s/n, 18071 Granada, Spain; 5 Department of Analytical Chemistry and Food Science and Technology, University of Castilla-La Mancha, Ronda de Calatrava, 7, 13071 Ciudad Real, Spain; 6 Regional Institute for Applied Scientific Research (IRICA), Area of Food Science, University of Castilla-La Mancha, Avenida Camilo Jose Cela, 10, 13071 Ciudad Real, Spain

**Keywords:** Salicornia, stroke, neuroprotection, polyphenol, ischemia

## Abstract

Strokes are the second most common cause of death worldwide and a leading cause of disability. Regular consumption of polyphenols has been shown to reduce the risk of suffering a cardiovascular event. For this reason, we have investigated the protective effect of *Salicornia ramosissima*, a seasonal halophyte that synthetizes high amounts of bioactive compounds, including polyphenols, in response to environmental stress. Aqueous, hydroalcoholic, and ethanolic extracts were prepared to investigate if dietary supplementation prior to ischemic challenge can prevent subsequent damage using two animal models. First, we screened the protective effect against hypoxia–reoxygenation in *Drosophila melanogaster* and observed that both ethanolic and hydroalcoholic extracts protected flies from the deleterious effects of hypoxia. Second, we confirmed the protective effect of *S. ramosissima* ethanolic extract against brain ischemia using the transient middle cerebral artery occlusion mice model. Four weeks of oral supplementation with the ethanolic extract before artery occlusion reduced infarct volume and lowered the plasma levels of the DNA peroxidant product 8-hydroxydeoxyguanosine. Phytochemical profiling of *S. ramosissima* ethanolic extract revealed 50 compounds. Thus, it represents a valuable source of bioactive compounds that show promising disease-modifying activities and could be further developed as an effective food supplement for the prevention or treatment of neurovascular disorders.

## 1. Introduction

Stroke is the most common neurovascular disease, representing the second most common cause of death and the third most common cause of disability worldwide, according to the World Health Organization. Ischemic stroke encompasses different mechanisms of vascular occlusion and represent around 80% of all stroke cases [1]. Changes in brain-regional blood flow deprive this highly demanding tissue of oxygen and nutrients, leading to a cascade of metabolic and molecular processes resulting in significant neuronal death shortly after ischemia [2]. More than a hundred drug candidates for stroke treatment have been tested in clinical trials [3], but mechanical thrombectomy and intravenous thrombolysis using tissue plasminogen activator (t-PA) or tenecteplase (TNK) are still the only approved medical therapies for ischemic stroke and, unfortunately, no neuroprotectant drug has ever been shown to be useful in stroke patients [4].

Epidemiologic data show that the incidence of stroke has fallen in high-income countries with the improvement in the control of risk factors, such as hypertension [5], hyperlipidaemia, diabetes [6], or atrial fibrillation [7]. Most risk factors are very prevalent and, potentially, treatable. Therefore, a suggested strategy to prevent or reduce ischemic brain injury is identifying populations at high vascular risk, such as patients having coexistence of several vascular risk factors or silent brain infarcts, or those undergoing carotid artery or heart surgery, to whom neuroprotective therapies could be administered before a stroke might occur [8]. In this sense, nutraceuticals have been reported to reduce stroke risk and improve recovery [9] and, if necessary, can be administered for years as prophylactic treatments depending on their safety profile.

*Salicornia ramosissima*, commonly known as glasswort, is a seasonal Mediterranean saltmarsh plant that, in response to saline stress and UV radiation, synthetizes bioactive compounds, such as polyphenols [10], which are of great interest for their therapeutic effects in cardiovascular diseases [11]. Green aerial parts of different Salicornia species have been used for a long time as food sources in salads, pickles, beverages and, more recently, as green salt. Historically, Salicornia has also been used for non-edible purposes in traditional medicine as a remedy for constipation, infections, or diabetes, among other conditions [12]. More recently, a *S. fragilis* extract rich in polyphenols has been attributed with antioxidant activities [13]. Moreover, *S. europaea* extracts have been reported to ameliorate hypertension and vascular dysfunction induced by high salt consumption in vivo [14], as well as reduce hyperplasia during vascular remodeling [15]. In addition, oral administration of a desalted *S. europaea* extract prior to scopolamine-induced amnesia protected mice from cognitive impairment and promoted neurogenesis [16].

Among phytochemicals previously found in *S. ramosissima*, phenolic compounds, such as flavonoids derivatives (luteolin or quercetin glucosides), have been reported to be relevant compounds in the prevention of several disorders, such as cancer, hypertension, or neurodegeneration, taking action in the regulation of several metabolic pathways or decreasing cancer cell growth [17,18]. Moreover, phenolic acids, such as tungtungmadic acid, have been reported to be hepatoprotective by reducing necrosis and inflammation of the liver in a mice model of hepatic fibrosis induced by carbon tetrachloride [19].

Although accumulating evidence supports the therapeutic potential of Salicornia’s phytochemical profile, there are no reports investigating its role against hypoxia or ischemia. In the present study, we have evaluated whether a *S. ramosissima* ethanolic extract, a powerful source of polyphenols, protects from the deleterious effects induced by ischemia.

## 2. Materials and Methods

### 2.1. Extracts Preparation Procedure

Freshly collected *S. ramosissima* aerial parts were freeze-dried until complete loss of moisture and subsequently ground to facilitate the extraction process. Plant materials were subjected to solid–liquid extraction (plant to solvent ratio 1:10, m:v (g:mL)) by using ethanol (Salicornia ethanolic extract (S-EE)), hydroalcoholic mixture (50:50; ethanol: water; *v*:*v*) (Salicornia hydroalcoholic extract (S-HE)), or water (Salicornia aqueous extract (S-AE)) for 2 h at 45 °C and at a stirring speed of 170 rpm. Then, supernatant was collected, centrifuged, and filtered (0.45 μm). Extracts were evaporated under vacuum and dry extracts were kept light-protected at −20 °C until later use.

### 2.2. Determination of Total Phenolic Content

The total content of phenolic compounds was assessed using the Folin–Ciocalteu method. Briefly, Salicornia extract stocks (20 mg/mL in 20:80 *v*/*v* ethanol:water) were diluted to 1 mg/mL in water. Then, 30 μL of each sample (1 mg/mL) or standard (Gallic acid) were mixed in triplicates with 30 μL of Folin–Ciocalteu reagent (1:10 dilution in water) in a 96-well plate and incubated at room temperature for 5 min. Finally, 5% sodium carbonate (*w*/*v*, 240 μL) was added and the plate was incubated for 1.5 h at 30 °C. Absorbance was measured at 760 nm using a CLARIOstar spectrophotometer (BMG Labtech, Ortenberg, Germany). Total phenolic content was calculated using a Gallic acid calibration curve within a range of 0–100 μg/mL. Results were expressed as mg Gallic acid equivalents (GAE)/g of dry extract.

### 2.3. Phytochemical Profile of S-EE by HPLC-ESI-QTOF-MS Methodology

The sample was reconstituted from the dry extract at a concentration of 5 mg/mL and filtered before high-performance liquid chromatography coupled to electrospray ionization and quadrupole time-of-flight mass spectrometry (HPLC-ESI-QTOF-MS) analysis. The analysis was carried out in a high-pressure liquid chromatography platform (Agilent 1290 HPLC, Agilent Technologies, Palo Alto, CA, USA) coupled to mass spectrometry using a quadrupole time-of-flight analyzer (Agilent 6545 QTOF Ultra High Definition, Agilent Technologies, Palo Alto, CA, USA).

The chromatographic method was carried out in reverse phase with a C18 column (ACQUITY UPLC BEH, 1.7 µm, 2.1 mm, 150 mm, 130 Å, Waters Corporation). The column temperature was fixed at 60 °C. As mobile phases, water with 0.1% of formic acid (A) and acetonitrile (B) were used. The following mobile phase gradient was used for the separation: 0 min [A:B 95/5], 5 min [A:B 90/10], 18 min [A:B 15/85], 24 min [A:B 0/100], 25.50 min [A:B 0/100], 26.50 min [A:B 95/5], and 32.50 min [A:B 95/5]. The mobile phase flow rate was 0.4 mL/min and the injection volume of the sample was 5 µL.

MS acquisition was performed in negative electrospray ionization mode (ESI-) with a mass/charge ratio range between 50 and 1200 *m*/*z*. Two reference masses—purine (*m*/*z* 121.0509) and HP-921 (*m*/*z* 922.0098)—were used for the calibration of all *m*/*z* values by means of a continuous infusion. S-EE was analyzed in full scan acquisition mode with a scan rate of 3 spectra/sec. The MS data were acquired in centroid mode. Other parameters were as follows: gas temperature 200 °C; gas flow 10 L/min; nebulizer 20 psig; sheath gas temperature 350 °C; sheath gas flow 12 L/min, VCap 4000 V; and Nozzle Voltage 500 V. The acquired MS data were converted to an mzML format using MSConverter (Proteowizard) and then processed using the mzMine 2.53 software. The compounds were annotated according to the comparison of the experimental data (Retention time, *m*/*z*, molecular formula) with those found in the literature and databases.

### 2.4. Drosophila Treatments and Exposure to Hypoxia

*D. melanogaster* (Oregon R strain) flies were kindly provided by Dr. Luisma Escudero. Flies were reared on a standard medium in plastic vials at a constant temperature and humidity (25 °C; 40% humidity) under a 12 h/12 h light/dark cycle. Flies were sexed under CO_2_ anesthesia 1–3 days after emergence and males were selected and placed in plastic vials containing Nutrifly instant formulation media (Genesee Scientific, San Diego, CA, USA). Each experiment was performed in triplicates (3 drosophila vials per condition containing 10–15 male flies each). The treated groups received instant formulation prepared in water containing Salicornia extracts at the indicated concentrations for 5 days. The control and hypoxia groups were kept on instant formulation media prepared in vehicle (0.5% ethanol in water). Treatment media were refreshed once during the experimental procedure.

To study any possible toxic effect of Salicornia treatments, male survival was assessed daily during the treatment length in normoxic conditions. After 5 days of treatment, vials containing 10–15 male flies were subjected to 1% O_2_ for 2.5 h using a Coy O_2_ Control In Vitro Glove Box (Coy, Grass Lake, MI, USA). N_2_ was used to displace the atmospheric gas, and temperature (25 °C) and humidity (30–40%) were controlled during the hypoxia challenge. Then, flies were allowed to recover in normoxia maintained in standard medium. Mortality rate in each tube was assessed.

### 2.5. Drosophila Locomotor Activity Assay

To study the impact of hypoxia on locomotor activity, each group of flies was transferred into a 25 mm empty polycarbonate vial and placed in the Drosophila Activity Monitoring (DAM) system (LAM25H-3, Trikinetics Inc., Waltham, MA, USA). Locomotor activity was assessed following the hypoxia exposure by registering the infrared light beam crossing at 3 different heights vertically on each tube. The DAMSystem3 Data Collection Software was used for data acquisition every 5 s. Raw data were then processed using the FileScan Software to ensure that all data records are complete and converted to 30 min intervals for analysis. Live flies were counted at the beginning and the end of the assessment period and mean beam crosses every 30 min was calculated.

### 2.6. Animals and Transient Focal Cerebral Ischemia Model

All animal care and experimental procedures were performed in accordance with the European Union guideline and approved by the Animal Research Ethic Committee of the Vall d’Hebrón Institute (protocol number 03/19) and the Regional Committee for Animal Experimentation. Six-week-old C57BL/6J male mice (Janvier Laboratories, Le Genest-Saint-Isle, France) were housed under a 12 h light–dark cycle at a temperature of 22 °C and allowed to consume food and tap water ad libitum throughout the study period. Thirty mice were randomly assigned into two groups, and per os (p.o.) administered with 100 mg/kg of S-EE or vehicle (20% ethanol solution in water) for 28 days before surgery. All animals were weighed daily during the experimental procedure.

A 90 min transient proximal middle cerebral artery occlusion (t-pMCAo) model was performed blindly by introducing an intraluminal filament (Doccol 602256PK10Re) as described previously [20]. Only animals presenting a reduction in cerebral blood flow of 80% after filament introduction and a recovery of 75% after filament removal were included in the study. Overall, 10 mice were excluded because of treatment or surgery failure. Finally, 10 mice per group were included in this study.

Then, 24 h after the surgery, animals received deep anesthesia and blood samples were collected in EDTA anticoagulated tubes, centrifuged at 1000× *g* for 15 min and plasma was collected and stored in −80 °C until use. Animals were then transcardially perfused with cold saline and brains were quickly collected for analysis.

### 2.7. Infarct Volume Assessment

Immediately after collection, the mice brains were sectioned into 1 mm thick slices and stained using 2.5% 2,3,5-triphenyl-2H-tetrazolium chloride (TTC; Sigma-Aldrich, Saint Louis, MO, USA). TTC images were captured using a CanoScan 4200F scanner (Canon, Tokyo, Japan) and quantified with Image J software. The white ischemic lesion was calculated blindly as a percentage of infarct volume of the hemisphere ipsilateral to the lesion.

### 2.8. Functional and Neurological Assessment

Grip strength test was performed to assess the peak forelimb force using a computerized grip strength meter (Harvard Apparatus, Cambridge, MA, USA) one day before the operation, and then repeated at 24 h post-surgery, as described previously [21]. At 24 h after occlusion, as well as the grip test, a latency to full body movement test and a 39-point neurological score were also performed blindly on each mouse [22,23,24].

For the latency–movement test, more than 3 min was recorded as 180 s. Both general deficits (total score 13) and focal deficits (total score 26) were evaluated using a 39-point neurological score method, in which a higher score represents a worse outcome.

### 2.9. Preparation of Brain Homogenates

Brain slices were split in two hemispheres: the ipsilateral and contralateral hemisphere. After 1 mL of PBS was added to each sample, brain samples were homogenized with a sonicator and centrifuged at 8000× *g* for 10 min. The supernatants were collected and stored at −80 °C until analysis. Bicinchoninic acid (BCA) assay was performed to detect the total protein amount in brain homogenate. The molecule amount in the brains was calibrated using the total protein amount of each sample.

### 2.10. Quantification of Brain and Plasma Antioxidation Markers

Antioxidation-related molecules, including antioxidants, lipid peroxidation and DNA oxidation products, were analyzed in both plasma and brain homogenate samples. Sample size was reduced to n = 6–8 (2 out of 10 animals in vehicle were not included in the ELISA measurements due to the maximal measure capability in one 96-well plate and one plasma sample of the S-EE-treated mice did not reach detection limits for the measured parameters). Experiments were performed following the instructions provided by the commercially available ELISA kits: Thioredoxine (TRX, Abx254796, Abbexa, Cambridge, UK), 4-Hydroxynonenal (4-HNE, MOFI01251, AssayGenie, Dublin, Ireland), and 8-hydroxydeoxyguanosine (8-OHdG, CSB-E10527m, Cusabio, Hertfordshire, UK). The detection sensitivity of TRX, 4-HNE, and 8-OHdG is 37.5 pg/mL, 18.75 pg/mL, and 0.195 pg/mL, respectively. The intra- and inter-assay coefficients of variation (CVs) of TRX, 4-HNE and 8-OHdG were all < 10%. All standards and samples (except TRX measurement) were tested in duplicate wells. Values showing CV value of OD > 20% were excluded for the analysis. Data reflecting molecule amount in brain homogenates were then calibrated with the total protein amount in each sample and represented as molecule amount per nanogram (ng) total protein. To compare the difference in molecule expression in two groups, the percentage in the ipsilateral hemisphere versus the contralateral hemisphere was introduced.

### 2.11. Statistics

Data are expressed as mean ± standard deviation (s.d.). Data were checked for normal distribution using the Kolmogorov–Smirnov test. For Drosophila assays, groups were compared using one-way ANOVA analysis followed by Dunnett´s multiple comparisons test. For mice experiments, data were compared using unpaired t-tests. Differences in mortality between groups in mice experiments were assessed using the chi-square test. All statistical analysis was performed using GraphPad Prism v.8 and significance was set as *p* < 0.05.

## 3. Results

### 3.1. Content of Phenolic Compounds in S. ramosissima Extracts

It is known that halophytes are a great source of biological compounds [25]. The total phenolic content in Salicornia extracts was determined using the Folin–Ciocalteu assay. The content of polyphenols varied considerably among the three extracted fractions, ranging from 7 to 46.3 mg GAE/g (Table 1). The S-EE showed the highest phenolic content (46.3 mg GAE/g) followed by the S-HE extract (23.5 mg GAE/g). In contrast, the S-AE was less rich in phenolic compounds (7 mg GAE/g).

### 3.2. Tentative Characterization of S-EE by HPLC-ESI-QTOF-MS

Following the non-targeted method for the annotation of the phytochemical profile of S-EE, fifty compounds have been detected. Table 2 shows the analytical information of each compound (retention time, *m*/*z*, molecular formula, and proposed compound) and the previous data found in the literature. The major abundance of bioactive compounds in this extract is associated with caffeoylquinic acid derivatives, flavonoids, and fatty acids.

### 3.3. Effect of Supplementation with S. ramosissima Extracts on Drosophila Melanogaster Performance after Severe Hypoxia

**Figure 1:**
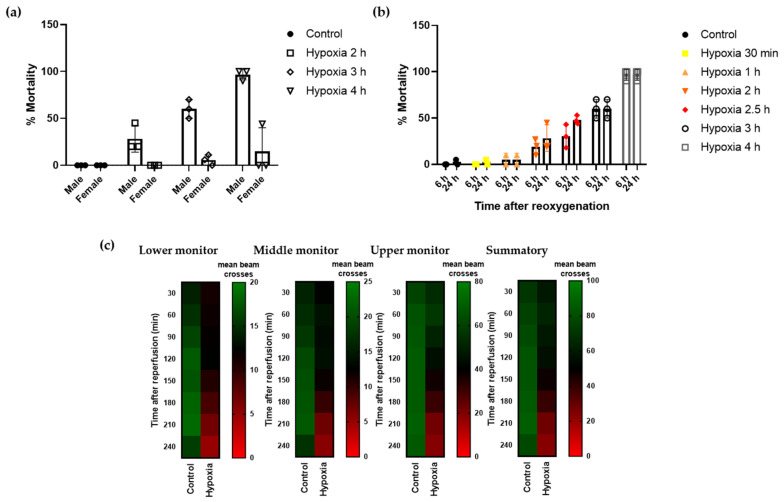
Mortality rate and locomotor activity of Drosophila after hypoxia. Flies were submitted to 1% O_2_ for different time periods followed by reoxygenation (n = 3 experiments, 90 individuals per condition). (**a**) Mortality rate of male and female flies 24 h after exposure to different durations of hypoxia. (**b**) Mortality rate of male flies 6 and 24 h after hypoxia exposure. (**c**) Heatmap displaying the locomotor activity of male flies following 2.5 h of hypoxia or normoxia. Flies were transferred into the DAM system immediately after hypoxia and activity was recorded for 240 min after reperfusion. Each cell shows the mean beam crosses per fly.

Drosophila melanogaster has been widely used as a model system in neurodegeneration for its limited need for resources and the high degree of conservation, including the entire hypoxia cascade [37]. In initial assays, we observed dimorphic response to the detrimental effects of hypoxia, with males being more vulnerable to hypoxia/reperfusion injury than females (Figure 1a). Therefore, to standardize our experiments, we conducted hypoxia using male flies only. To investigate the influence of severe hypoxia on Drosophila males, flies were subjected to hypoxic stress under controlled conditions (1% O_2_; 25 °C; 30–40% relative humidity) at increasing time durations. As shown, increasing the time of severe hypoxia exposure resulted in higher mortality rates, with 4 h hypoxia resulting in almost 100% mortality (Figure 1b). Therefore, we selected the half-lethal hypoxic stimulus of 2.5 h to define a reliable protocol to study the effect of the treatments on mortality rates. Next, we confirmed that flies exposed to 2.5 h hypoxia exhibited a locomotor activity deficit compared to control flies. We monitored the number of beam crosses of each experimental group using the DAM system and observed that control flies moved uniformly over time and the number of beam crosses were higher at the upper monitor, as expected given the innate locomotor behavior in Drosophila. Flies undergoing hypoxia showed decreased locomotor activity, indicated by fewer beam crosses in all the monitors of the unit (lower, middle, and upper monitor). Moreover, locomotor activity after hypoxia was further reduced over time, especially after 120–150 min of reoxygenation, revealing a delayed effect of hypoxic injury on flies’ behavior (Figure 1c).

**Figure 2:**
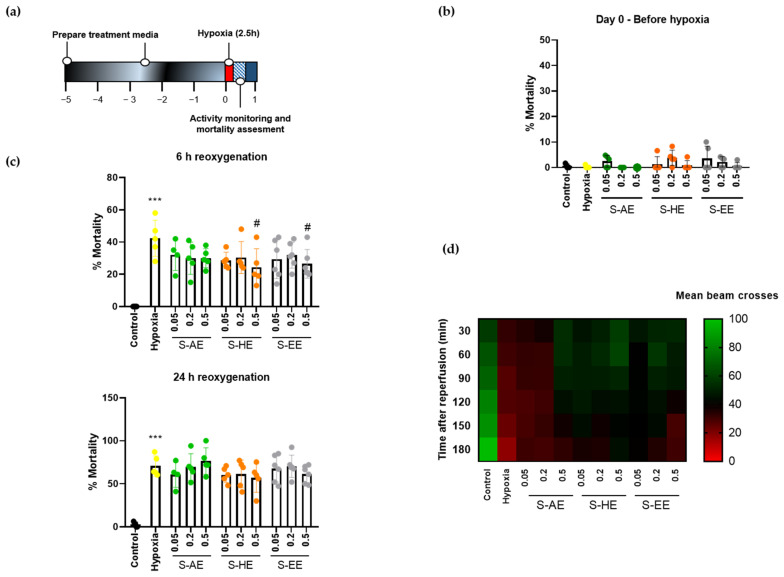
Effect of different concentrations of S-AE, S-HE, and S-EE pre-treatment (mg/mL) on flies exposed to acute hypoxia. (**a**) Schematic illustration of the hypoxia protocol in Drosophila males. (**b**) Mortality rate assessment after 5 days of treatment in normoxia (n = 5 experiments; 150–180 individuals per condition). (**c**) Mortality rate of control and treated flies 6 and 24 h after hypoxia exposure (n = 5 experiments; 136–181 individuals per condition). (**d**) Heatmap displaying the locomotor activity of treated flies (n = 3 experiments; 79–90 individuals per condition). Flies were transferred into the DAM system immediately after hypoxia and activity was recorded for 180 min after reoxygenation. Each cell shows the mean beam crosses per fly. Data were analyzed using one-way ANOVA test followed by Dunnett´s multiple comparisons test *** *p* < 0.001 vs. control; # *p* < 0.05 vs. hypoxia.

Then, we studied the effect of the three Salicornia extracts on hypoxia performance of Drosophila. Flies were treated for 5 days with different doses of S-AE, S-HE, or S-EE and subjected to severe hypoxia for 2.5 h followed by reoxygenation (Figure 2a). No signs of toxicity were observed for any of the extracts, since mortality rates after 5 days of treatment at the highest doses under normoxic conditions were similar to the control group (Figure 2b). Treatment with S-AE for 5 days did not protect flies from hypoxia-induced mortality. On the contrary, S-HE and S-EE at their highest dose (0.5 mg/mL) significantly reduced mortality rate after hypoxia (Figure 2c, upper panel). However, the protective effect of both extracts was lost 24 h after reoxygenation in standard non-supplemented media (Figure 2c, bottom panel). Finally, the effect of Salicornia extracts on hypoxia-induced locomotor deficit was studied after reperfusion. As expected, hypoxia-exposed flies showed a decline in locomotor activity. On the other hand, pre-treatment with S-AE at the highest dose, as well as the three different doses of S-HE and S-EE greatly improved general locomotor activity after hypoxia exposure and postponed the locomotor decline compared to the hypoxia group (Figure 2d).

### 3.4. Oral Supplementation with S-EE Prevented Brain Damage after Experimental Stroke in Mice

**Figure 3:**
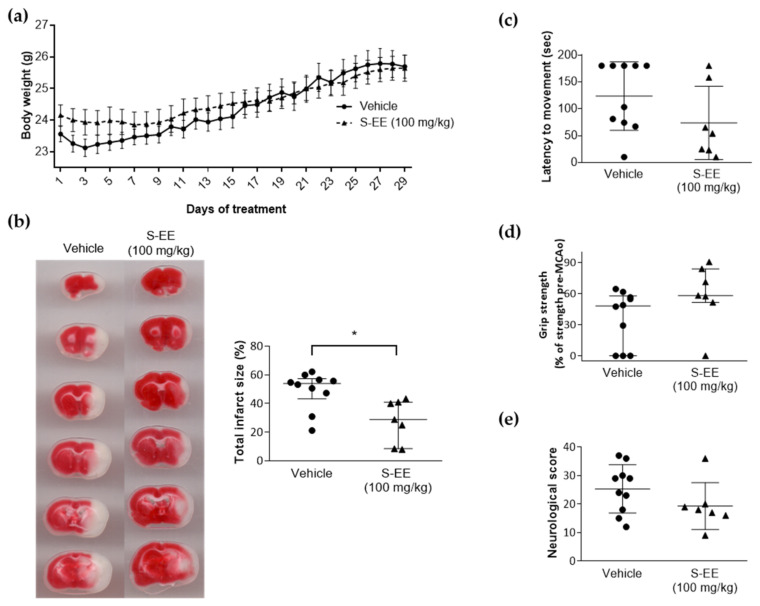
Effect of oral supplementation with S-EE for 28 days on experimental strokes in mice. (**a**) Daily bodyweight monitoring after 28 days of treatment with S-EE or vehicle. (**b**) Representative images of TTC brain staining and quantification of percentage of infarct volume 24 h after t-pMCAo. Neurological and functional evaluation were performed 24 h after surgery including latency–movement test (**c**), grip strength test (**d**) and neurological score (**e**). Sample size: vehicle n = 10; S-EE n = 7. Data were analyzed using Student’s t-test. * *p* < 0.05 vs. vehicle.

We investigated whether the protective effect observed for S-EE, the extract richest in polyphenols, was translated to mice brain ischemia. To this end, mice were given oral gavage supplementation with S-EE (100 mg/kg) or vehicle for 28 days prior to the induction of experimental stroke. No signs of toxicity or significant weight loss were observed for S-EE treatment during the experimental period (Figure 3a). Three out of ten mice treated with the extract died after the surgery before scarification, while no mortality was found in animals treated with vehicle. No significant difference was found between groups (*p* > 0.1, Chi-square test). As shown in brain images, oral supplementation with S-EE significantly reduced brain infarct volume after 90 min of t-pMCAo.

To determine the effect of S-EE on functional outcomes, a latency–movement test and a grip test were performed, as well as a neurological scale for general and focal deficit evaluation. Compared to the vehicle group, animals treated with S-EE showed a tendency towards reduced time of latency to movement and increased grip strength (Figure 3c,d). No difference was found regarding neurological score (Figure 3e).

### 3.5. Effect of S-EE Supplementation Oxidative Stress Markers in Plasma and the Brain

**Figure 4:**
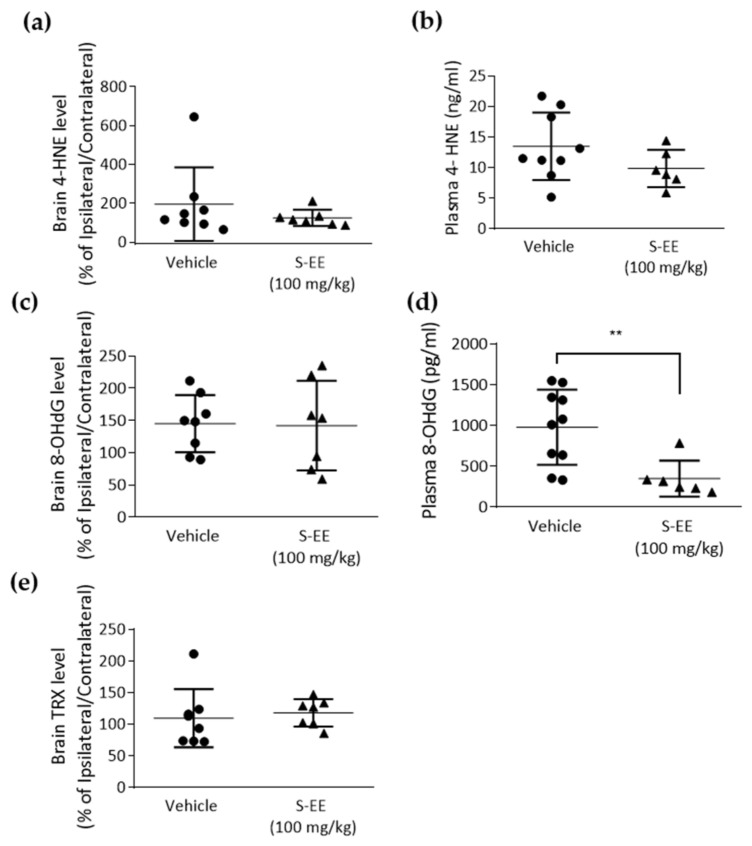
Oxidative stress markers measurement in mice plasma and brain tissue. Samples were collected 24 h after t-pMCAo and analyzed for 4-HNE (**a**,**b**), 8-OHdG (**c**,**d**) or TRX (**e**) levels. Data were analyzed using Student’s t-test. Sample size: brain samples (vehicle n = 8; S-EE n = 7), plasma samples (vehicle n = 8; S-E E n = 6). ** *p* < 0.01 vs. vehicle.

The brain is characterized by a high requirement of oxygen and relatively low antioxidant capacity, so it is very vulnerable to ischemia. A major role of oxidative stress in stroke injury has been suggested and animal models of the disease have shown decreased brain and plasma antioxidant capacity [38]. Therefore, we analyzed plasma and brain levels of different tissue oxidation markers. First, we measured the level of the lipid peroxidant product 4-hydroxynonenal (4-HNE) in the brain and found no effect in S-EE-treated mice compared to vehicle (Figure 4a). When measuring 4-HNE in plasma, we observed a trend of a reduced level of 4-HNE after S-EE supplementation, although it did not reach statistical significance (Figure 4b). We also assessed 8-hydroxydeoxyguanosine (8-OHdG), an oxidized nucleoside of DNA. Again, no significant changes were observed in the brain (Figure 4c), while plasma levels of 8-OHdG were significantly reduced when mice were treated with S-EE for 28 days prior to t-pMCAo (Figure 4d). Finally, levels of thioredoxin (TRX), a redox-regulating protein with antioxidant activity, were measured in brain tissue, but no effect was observed in S-EE-treated mice (Figure 4e).

## 4. Discussion

Current evidence suggests the presence of important bioactive compounds in halophyte plants. Here, we investigated *S. ramosissima* as a source of polyphenols for the treatment of brain ischemia. The preparation of aqueous, hydroalcoholic, and ethanolic *S. ramosissima* extracts proved that ethanol concentration in the extraction solvent affected the recovery of phenolic compounds, with S-EE extraction being the most suitable for the highest recovery of polyphenols.

To our knowledge, this is the first study reporting the neuroprotective effect of *S. ramosissima* against ischemic brain damage. The three extracts were screened for protective potential using a Drosophila model of hypoxia–reoxygenation. We observed that pre-treatment with S-HE and S-EE reduced hypoxia-induced mortality and locomotor impairment. Moreover, we showed here that oral supplementation with S-EE (100 mg/kg) for four weeks before ischemia protected mice brains from experimental strokes, as shown by a significant reduction in t-pMCAo-induced infarction, a tendency to improve functional outcome and a reduction in plasma 8-OHdG levels.

Salicornia has been extensively studied in Chinese medicine and numerous reports have attributed this halophyte antioxidant [39,40], antithrombotic [41], anti-inflammatory, or analgesic [42] properties. In fact, a *S. herbacea* ethanolic extract was found to be neuroprotective in vitro through the induction of antioxidant defense enzymes [17] and an ethyl acetate extract of *S. europaea* protected mice from a Parkinson´s disease-like model by regulating proinflammatory molecules [43]. In our study, dietetic supplementation with S-EE protected fruit flies from global hypoxia so a beneficial effect against ischemia in other tissues different from the brain may also be plausible. Moreover, using the t-pMCAo model in mice, a systemic effect of S-EE was also observed, as shown by a reduction in plasma levels of the oxidative stress markers, 4-HNE and 8-OHdG, 24 h after reperfusion.

Salicornia extracts represent a complex source of biomolecules with neuroprotective or antioxidant activities [10]. As expected by previous reports [26,27,28,29,30], we found many different compounds that can be responsible for the therapeutic effect of S-EE, mainly including caffeoylquinic acid derivatives, flavonoids, and fatty acids. Although the exact therapeutic mechanisms of S-EE were not revealed in this study, several phytochemicals present in the plant might have contributed to the protective effect in combination. Among them, quercetin administration prior to the onset of ischemia has been reported in several studies to reduce brain infarct volume and neuronal loss, and to improve neurological outcome [44,45,46]. Another major component of *S. ramosissima*, caffeic acid, ameliorated neurological dysfunction and reduced infarct volume after focal and global ischemia [47,48]. In addition, the presence of caffeoylquinic acid and its derivatives in *Artemisia princeps Pampanini* extract showed a neuroprotective effect on PC-12 cells under the insult of amyloid ß peptide by reducing oxidative stress [49]. Several works have reported that ferulic acid, a derivative of cinnamic acid also present in S-EE, protects against experimental cerebral ischemia-reperfusion in vivo, reducing brain infarct through antioxidant mechanisms [50,51]. Interestingly, we found cannabidiolic acid (CBDA) to be present in S-EE. CBDA is a less stable carboxylated precursor of CBD, which has been broadly studied as a neuroprotectant against many conditions, including brain ischemia [52]. Additionally, the main fatty acids found in our Salicornia extracts, linoleic and linolenic acids, are widely known for their role in cardiovascular protection [53,54,55].

As suggested by the Stroke Therapy Academic Industry Roundtable (STAIR) [56], our data should be replicated in new studies with the inclusion of female, aged animals and comorbid conditions, such as hypertension, diabetes, and hypercholesterolemia in order to increase the translational potential of the treatment, but also to identify feasible therapeutic windows. In our experimental approach, S-EE reduced flies’ death at 6 h after reoxygenation when given before hypoxia. However, the protective effect was lost at 24 h after reoxygenation, suggesting that prophylactic treatment is not enough for improving long-term outcomes, and, therefore, treatment might need to be extended and continued after hypoxia. In mice, a neuroprotective effect was also observed when S-EE treatment was performed before ischemia onset. Despite this strategy being challenging to translate into the clinic, many strokes can be prevented through the control of risk factors. In this sense, a *S. herbacea* extract was shown to control hyperlipidemia in diabetic mice through inhibition of pancreatic lipase [57]. Moreover, other authors have suggested that Salicornia extracts would be a useful dietary supplement to avoid abnormal vascular events that could precede stroke, such as vascular dysfunction and remodeling [14,15]. For this reason, Salicornia is being studied as a salt substitute in fermented food [58].

The current interest in nutraceuticals from both industry and consumers is notably rising due to their safety and therapeutic effects. A randomized controlled trial has recently shown that oral administration of desalted *S.europaea* ethanolic extract for 12 weeks is well tolerated by subjects complaining of memory dysfunction [59]. In agreement with this, some major constituents described in Salicornia extracts are marketed as ingredients of dietary supplements for different health conditions [60], including quercetin [61], apigenin [62], or chrysin [63]. In this respect, our group is currently evaluating the long-term safety and tolerability of S-EE in healthy volunteers.

Taken together, the results shown in this study highlight the protective effect of S-EE against hypoxia/ischemia in both fruit flies and mice models. Considering the rich composition of bioactive substances described in Salicornia and their safety profile, S-EE might serve as a potential candidate for the long-term prevention of neurovascular disease. Further studies are needed to elucidate the mechanism of action of isolated compounds and the synergic effects of S-EE components underlying its protective effect against ischemia.

## Figures and Tables

**Table 1:** Total phenolic content in *S. ramosissima* extracts. Results are expressed as mean ± standard deviation of five independent experiments.

	mg GAE/g DW	s.d.
S-HE	24	±2
S-AE	7	±2
S-EE	46	±3

GAE: gallic acid equivalent; DW: dry weight.

**Table 2:** Annotated compounds in S-EE by HPLC-ESI-QTOF-MS.

RT	*m*/*z*	Molecular Formula	Proposed Compounds	References
1.00	157.0368	C4H6N4O3	Allantoin	
1.03	191.0550	C7H12O6	Quinic acid	[26]
1.09	191.0223	C10H8O4	Scopoletin	[27]
6.33	355.1033	C16H20O9	Hydrocaffeoylquinic acid	[26]
6.52	431.1932	C20H32O10	Sacranoside A	
6.85	433.2087	C20H34O10	Benzyl trilactosylthreitol	
6.99	353.0881	C16H18O9	Neochlorogenic acid	[26]
7.21	165.0556	C9H10O3	Apocynin (caffeyl alcohol)	[28]
9.00	193.0513	C10H10O4	Ferulic acid	[26]
9.08	163.0393	C9H8O3	Coumaric acid	[26]
9.44	305.0703	C15H14O7	(Epi)gallocatechin	[26]
9.60	609.1458	C27H30O16	Quercetin-rhamnosyl-hexoside	[26]
9.67	417.2131	C20H34O9	Maryal	
9.85	463.0874	C21H20O12	Quercetin glucoside	[26]
9.86	517.1358	C25H26O12	Tungtungmadic acid isomer 1	[29]
10.08	517.1352	C25H26O12	Tungtungmadic acid isomer 2	[29]
10.16	515.1208	C25H24O12	Dicaffeoylquinic acid isomer 1	[30]
10.24	549.0886	C24H22O15	Quercetin malonyglucoside	[26]
10.30	515.1206	C25H24O12	Dicaffeoylquinic acid isomer 2	[30]
10.34	477.1018	C22H22O12	Isorhamnetin glucopyranoside	[29]
10.51	517.1352	C25H26O12	Tungtungmadic acid isomer 3	[29]
10.61	515.1200	C25H24O12	Dicaffeoylquinic acid isomer 3	[30]
10.84	519.1158	C24H24O13	Luteolin glucosyllactate	
10.89	515.1224	C25H24O13	Dicaffeoylquinic acid isomer 4	[30]
11.69	793.4033	C42H66O14	Calenduloside G isomer 1	[30]
12.04	327.2182	C18H32O5	Trihydroxyoctadecadienoic acid	
12.52	329.2342	C18H34O5	Trihydroxyoctadecenoic acid	
13.25	793.4375	C42H66O14	Calenduloside G isomer 2	[30]
13.33	289.1119	-	Unknown 1	
13.99	293.1763	C17H26O4	Embelin	
14.83	721.3693	-	Unknown 2	
15.22	562.3167	C28H52O11	Glycoside muricatin	[31]
15.71	559.3143	C28H48O11	Dirhamnosyl linolenic acid isomer 1	
15.82	293.2124	C18H30O3	Colneleic acid	
15.91	559.3137	C28H48O11	Dirhamnosyl linolenic acid isomer 2	
16.32	540.3312	C36H46O4	Bakuchiol derivative	[32]
16.51	295.2280	C18H32O3	Coriolic acid	[33]
18.41	357.2075	C22H30O4	Cannabidiolic acid	
18.59	277.2173	C18H30O2	Linolenic acid	
18.93	997.5766	C52H86O18	Ginsenoside derivative	[34]
19.29	279.2331	C18H32O2	Linoleic acid	
19.83	835.5233	C46H76O13	Glycerolipid derivative	[35]
19.96	255.2331	C16H32O2	Palmitic Acid	
20.71	981.5809	C52H86O17	Spirastrellolide B	
21.39	758.5436	C45H76O9	Decanedioic acid derivative isomer 1	
21.52	758.5438	C45H76O9	Decanedioic acid derivative isomer 2	
22.01	959.5975	C50H88O17	DGDG(33:3)acetate	[36]
21.76	819.5277	C46H76O12	Salinomycin derivative	
22.60	431.3177	C29H52O2	Tricosylresorcinol	
23.11	797.5438	-	Unknown 4	

## Data Availability

All relevant data are provided within the manuscript.
